# Human Evolution and Osteoporosis-Related Spinal Fractures

**DOI:** 10.1371/journal.pone.0026658

**Published:** 2011-10-19

**Authors:** Meghan M. Cotter, David A. Loomis, Scott W. Simpson, Bruce Latimer, Christopher J. Hernandez

**Affiliations:** 1 Department of Anatomy, Case Western Reserve University School of Medicine, Cleveland, Ohio, United States of America; 2 Musculoskeletal Mechanics and Materials Laboratory, Department of Mechanical and Aerospace Engineering, Case Western Reserve University, Cleveland, Ohio, United States of America; 3 Center for Human Origins, Case Western Reserve University, Cleveland, Ohio, United States of America; 4 Department of Anthropology, Case Western Reserve University, Cleveland, Ohio, United States of America; 5 Sibley School of Mechanical and Aerospace Engineering, Cornell University, Ithaca, New York, United States of America; Ohio State University, United States of America

## Abstract

The field of evolutionary medicine examines the possibility that some diseases are the result of trade-offs made in human evolution. Spinal fractures are the most common osteoporosis-related fracture in humans, but are not observed in apes, even in cases of severe osteopenia. In humans, the development of osteoporosis is influenced by peak bone mass and strength in early adulthood as well as age-related bone loss. Here, we examine the structural differences in the vertebral bodies (the portion of the vertebra most commonly involved in osteoporosis-related fractures) between humans and apes before age-related bone loss occurs. Vertebrae from young adult humans and chimpanzees, gorillas, orangutans, and gibbons (T8 vertebrae, n = 8–14 per species, male and female, humans: 20–40 years of age) were examined to determine bone strength (using finite element models), bone morphology (external shape), and trabecular microarchitecture (micro-computed tomography). The vertebrae of young adult humans are not as strong as those from apes after accounting for body mass (p<0.01). Human vertebrae are larger in size (volume, cross-sectional area, height) than in apes with a similar body mass. Young adult human vertebrae have significantly lower trabecular bone volume fraction (0.26±0.04 in humans and 0.37±0.07 in apes, mean ± SD, p<0.01) and thinner vertebral shells than apes (after accounting for body mass, p<0.01). Since human vertebrae are more porous and weaker than those in apes in young adulthood (after accounting for bone mass), even modest amounts of age-related bone loss may lead to vertebral fracture in humans, while in apes, larger amounts of bone loss would be required before a vertebral fracture becomes likely. We present arguments that differences in vertebral bone size and shape associated with reduced bone strength in humans is linked to evolutionary adaptations associated with bipedalism.

## Introduction

Evolutionary medicine is a valuable perspective that utilizes evolutionary theory to understand the ultimate causation of disease [1]. While medical research concentrates on understanding the biomolecular cascade of events resulting in disease, a major goal of evolutionary medicine is to understand the occurrence, prevalence, and distribution of pathology by considering the balance between natural selection, the natural history of the disease and other needs for survival. A common theme in evolutionary medicine is that susceptibility to disease is an unintended consequence of otherwise advantageous evolutionary adaptations. Spontaneous fractures of the vertebral body (referred to here as vertebral fractures) are the most common osteoporosis-related fracture in humans [2]. In contrast, spontaneous vertebral fractures have not been reported in either wild or captive apes, even in individuals with severe osteopenia [3]–[7]. Based on this observation, it has been proposed that humans are susceptible to osteoporosis and osteoporosis-related fractures as a result of evolutionary adaptations [8]–[10], although it is not clear what aspects of vertebral structure differ between humans and apes.

Whether or not an individual develops osteoporosis is determined by peak bone mass at skeletal maturity and the amount of bone loss in later adulthood. While age-related bone loss and its causes (menopause, etc.) have been well described in humans, wild apes have also been shown to develop severe age-related bone loss, as indicated by low bone mineral density in femora and lumbar vertebrae (t-scores as low as -6.0, far below the t-score of -2.5 used in the diagnosis of osteoporosis in humans) [3], [6], [11], suggesting that differences in age-related bone loss alone may not explain differences in susceptibility to spinal fracture between humans and apes. Furthermore, the development of osteoporosis in humans is believed to be sensitive to peak bone mass at skeletal maturity, a trait that has a strong inheritable component in humans [12]–[14]. The current study therefore addresses the idea that spinal fracture is a consequence of evolutionary adaptations by examining bone morphology and strength in young adult humans and apes (referred to together as “hominoids”).

The extant hominoids are useful for examining the role of evolution in osteoporosis and osteoporosis-related fractures in that they are phylogenetically similar yet diverse in size and habitual locomotion. Hominoids all display primarily orthograde posture although locomotory habits are quite different; gibbons (*Hylobates lar*) exhibit brachiation, orangutans (*Pongo pygmaeus*) exhibit careful quadrumanus climbing and brachiation, chimpanzees (*Pan troglodytes*) and gorillas (*Gorilla gorilla*) exhibit knuckle-walking and humans (*Homo sapiens*) exhibit obligate bipedalism [15]. Additionally, the body mass range within hominoids is large, from 6–7 kg (*Hylobates*) to more than 150 kg (male *Gorilla*).

In humans, vertebral fractures associated with aging and osteoporosis are often not associated with falls or identifiable trauma and are highly correlated with vertebral bone strength [16], [17]. Since tissue material properties of bone are similar in closely related species, bone mass and structure are more likely to explain differences in bone strength among these species [18]. A number of characteristics of the vertebral body have been associated with fracture risk within humans including reduced bone volume fraction, increased degree of trabecular anisotropy [19], reduced vertebral shell thickness [20], and variability in bone density within the vertebral body [19], [21], [22]. While structural aspects of the vertebral body associated with fracture risk within humans have been well documented, only a few studies have examined those structural aspects across species. In our prior work we used rudimentary biomechanical modeling techniques to estimate differences in bone strength among these genera [23] and examined small regions of trabecular microarchitecture in human and ape vertebrae [24]. Here, we combine these two approaches to document biomechanics, size, and shape of vertebral bodies in humans and apes, using a more precise biomechanical modeling approach (finite element modeling) and examination of the internal microstructure of the entire vertebral body. The long-term goal of this research is to understand how susceptibility to musculoskeletal disorders has been influenced by human evolutionary history. In this work we use comparative analysis of bone strength and structure between young adult humans and apes. Specifically we: 1) determine differences in vertebral compressive strength among the species using quantitative computed tomography (QCT)-based finite element modeling; and 2) determine if differences in microarchitectural structure of the vertebral body explain any differences in whole bone strength among species.

## Materials and Methods

### Specimen Collection and Gross Morphology

**Figure 1:**
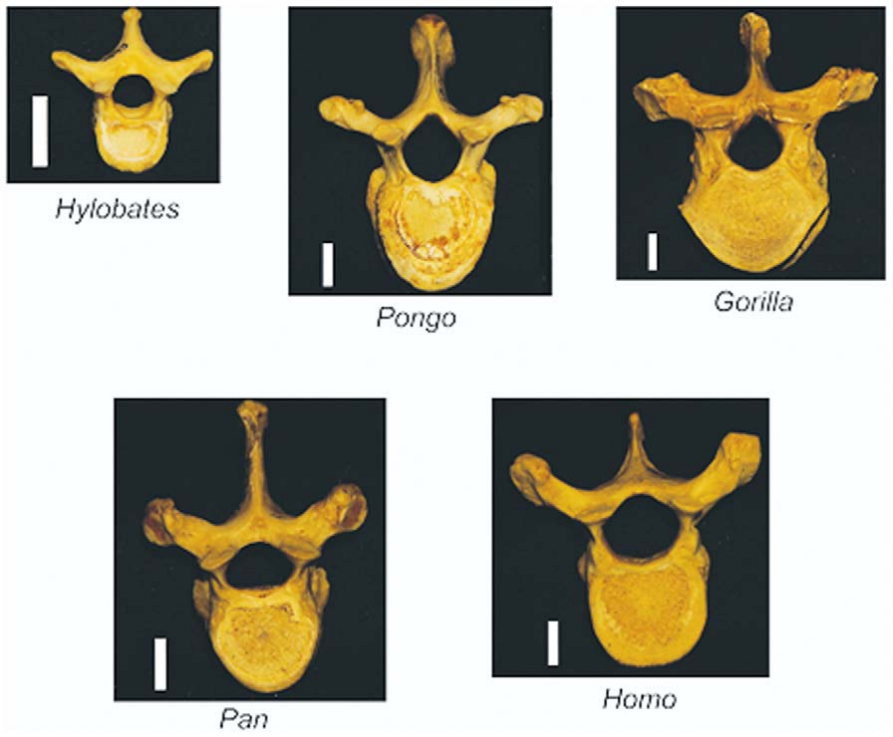
Specimens examined in the study. The study included thoracic vertebrae from five genera including *Hylobates* (gibbons), *Pongo* (orangutan), *Gorilla*, *Pan* (chimpanzee) and *Homo* (humans). The scale bar next to each representative specimen is one centimeter in length.

The study examined the eighth thoracic vertebra (T8) from young adult male and female wild-shot gibbons (*Hylobates lar*, n = 10), orangutans (*Pongo pygmaeus* and *Pongo abelii*, n = 8), western lowland gorillas (*Gorilla gorilla*, n = 10), chimpanzees (*Pan troglodytes*, n = 10) and modern humans (*Homo sapiens*, n = 14, 6 male, age 31.3±7.3 years, mean ± SD, range 20–40 years) (see Table 1, Figure 1). Specimens were from the Cleveland Museum of Natural History (Cleveland, OH, USA), the Field Museum of Natural History (Chicago, IL, USA) or the National Museum of Natural History (Washington D.C., USA) (the same study group was also examined in prior work [23]). Apes were confirmed as young adult if all epiphyses in the skeleton were closed and fused and by examination of tooth wear (all teeth emerged and molar wear was limited). Human specimens were selected from those with a sudden cause of death and known age at death to avoid individuals with altered bone morphology associated with chronic disease or age-related osteopenia. Individual body mass was estimated from measures of the femoral head (apes [25], humans [26], see [23] for details of body mass estimates). The T8 vertebra was chosen because it is one of the most common vertebrae to experience fracture in humans and it occupies the central kyphotic region of the thoracic spine in all of the species [27], [28]. Measures of vertebral body height (cranial-caudal distance on ventral surface), vertebral body width and depth (measured on the cranial endplate) were made of each specimen using calipers.

**Table 1:** Details of specimens used in finite element modeling and micro-computed tomography experiments are shown (All specimens used in finite element modeling, subset of specimens used in micro-computed tomography analysis noted in table).

Specimen ID*	Species	Sex	Ancestry	Age	Body Mass (kg)	Vertebra Height (mm)	Vertebra Cranial Cross-Sectional Area (mm^2^)	Bone Volume (cm^3^)	Trabecular BV/TV	Mean Shell Thickness (mm)
HTB3883	Hylobates lar				7.31	8.25	82.57	0.93	NM	NM
HTB3884	Hylobates lar				6.65	8.54	87.44	0.95	NM	NM
HTB3885	Hylobates lar				7.42	8.50	82.98	0.89	NM	NM
HTB3887	Hylobates lar				7.24	8.57	72.25	0.92	0.34	0.21
HTB3889	Hylobates lar				6.81	8.53	72.44	0.74	0.41	0.33
HTB3893	Hylobates lar				6.96	8.86	68.28	0.71	0.44	0.21
HTB3902	Hylobates lar				6.81	8.21	58.04	0.57	0.50	0.23
HTB3903	Hylobates lar				5.23	8.63	81.06	1.19	0.63	0.33
HTB3906	Hylobates lar				7.50	9.06	103.02	0.79	NM	NM
HTB3911	Hylobates lar				7.15	7.89	71.46	0.76	0.28	0.25
HTB1055	Pongo pygmaeus	F			47.83	14.52	350.02	6.33	0.29	0.61
FMNH33533	Pongo pygmaeus	F			49.83	16.12	465.20	8.90	NM	NM
FMNH33536	Pongo pygmaeus	F			41.58	15.33	273.56	5.81	NM	NM
NMNH49855	Pongo abelii	M			66.85	17.12	420.33	9.52	0.32	0.95
NMNH49859	Pongo abelii	M			59.96	17.31	518.62	9.31	0.28	1.01
NMNH145301	Pongo pygmaeus	M			86.08	17.60	467.96	12.09	0.31	0.93
NMNH145304	Pongo pygmaeus	M			73.53	17.42	651.25	13.26	0.31	1.02
NMNH145308	Pongo pygmaeus	F			44.75	15.39	537.21	8.88	0.31	0.70
HTB1710	Gorilla gorilla	F			80.91	15.48	394.42	8.03	0.38	0.75
HTB1765	Gorilla gorilla	F			79.39	15.77	548.65	8.83	0.28	0.63
HTB1797	Gorilla gorilla	M			172.77	18.45	891.47	20.61	0.39	0.76
HTB1798	Gorilla gorilla	F			88.83	15.26	532.38	8.95	0.30	0.96
HTB1859	Gorilla gorilla	M			163.05	20.72	784.51	21.77	0.27	1.04
HTB1992	Gorilla gorilla	F			94.59	14.63	475.98	8.66	0.35	0.76
HTB1997	Gorilla gorilla	F			77.35	15.39	483.89	8.92	0.40	0.78
HTB2741	Gorilla gorilla	M			201.40	19.95	979.69	23.74	0.399	1.12
HTB3391	Gorilla gorilla	M			140.50	18.21	678.19	14.15	0.45	0.87
HTB3404	Gorilla gorilla	M			141.13	19.64	857.27	19.20	0.41	0.92
HTB1719	Pan troglodytes	F			51.30	13.75	296.41	5.05	0.38	0.45
HTB1720	Pan troglodytes	F			36.32	14.24	328.87	4.75	0.35	0.57
HTB1722	Pan troglodytes	M			66.14	13.80	306.19	5.64	0.39	0.59
HTB1758	Pan troglodytes	M			61.86	14.91	383.96	7.47	0.35	0.78
HTB1766	Pan troglodytes	F			68.56	16.84	412.74	7.36	0.34	0.48
HTB1770	Pan troglodytes	F			37.55	13.54	284.18	4.48	0.41	0.53
HTB1880	Pan troglodytes	F			64.45	14.82	361.54	5.89	0.38	0.72
HTB2027	Pan troglodytes	M			51.91	14.79	370.51	6.53	0.34	0.57
HTB2072	Pan troglodytes	M			60.12	18.94	546.44	11.05	NM	NM
HTB3552	Pan troglodytes	M			54.37	15.42	345.13	6.42	0.39	0.69
HTH0074	Homo sapiens	M	B	35	63.90	19.69	781.40	16.82	NM	NM
HTH0243	Homo sapiens	F	W	40	56.67	18.64	637.43	13.08	NM	NM
HTH0249	Homo sapiens	F	W	40	56.60	20.06	494.89	11.95	0.21	0.38
HTH0339	Homo sapiens	F	W	38	70.59	19.76	553.72	12.87	0.24	0.54
HTH0439	Homo sapiens	F	B	35	58.31	17.50	523.11	8.95	NM	NM
HTH0561	Homo sapiens	F	B	25	56.36	18.79	424.31	10.08	0.27	0.64
HTH1208	Homo sapiens	F	B	23	57.39	18.45	426.86	9.76	NM	NM
HTH1785	Homo sapiens	F	B	32	58.19	19.15	544.60	12.38	0.24	0.44
HTH1787	Homo sapiens	F	B	40	58.30	18.89	520.16	10.57	NM	NM
HTH2085	Homo sapiens	M	B	23	70.31	21.13	646.05	14.87	0.34	0.62
HTH2104	Homo sapiens	M	B	20	69.81	21.80	495.61	11.63	0.27	0.66
HTH2169	Homo sapiens	F	B	24	67.25	21.15	563.96	13.63	0.26	0.55
HTH2193	Homo sapiens	M	A	25	65.66	19.10	557.91	11.76	0.20	0.36
HTH2206	Homo sapiens	M	W	31	80.41	21.53	618.83	16.33	0.27	0.53
HTH2831	Homo sapiens	M	W	38	73.18	21.36	712.03	16.33	0.23	0.53

*Specimens indicated by HTB or HTH are from the Physical Anthropology Collection at the Cleveland Museum of Natural History, Cleveland, Ohio. Specimens indicated by FMNH are from the Field Museum of Natural History, Chicago, Illinois. Specimens indicated by NMNH are from the Mammal Collection at the Smithsonian National Museum of Natural History, Washington, DC. Sex of Hylobates specimens was not available in museum records and could not be determined from skeletal analysis. Ancestry characterized at time of death (B - Black, W – White, A – Asian). NM – Not Measured because micro-computed tomography images were not taken of this specimen.

### Quantitative Computed Tomography and Biomechanical Modeling

Quantitative computed tomography scans were taken of all specimens along with a liquid calibration phantom (K_2_HPO_4_ calibration phantom, Mindways Software Inc.). Images were obtained with a 140 kV, 120 mA, signal with 0.75 mm slice thickness (Siemens Somatom 16, Malvern, PA, USA) or 0.625 mm slice thickness (Philips Brilliance 64, Andover, MA, USA, used on the two specimens from the Field Museum of Natural History). Two sample vertebrae, unaffiliated with the study, were scanned at both locations to ensure compatibility between scanners. Bones were submerged in a 20% ethanol solution to improve accuracy of QCT density values. A vacuum was applied at 30 in. Hg for 30 minutes prior to scanning to remove any air bubbles present in the bone cavities.

Computed tomography images were analyzed using custom software written for use with MATLAB (version 7.8.0, Mathworks Inc., Natick, MA, USA). Background signal associated with the ethanol solution was removed by subtracting a grayscale density value comparable to one standard deviation below the mean fluid value [29]. Images underwent Gaussian filtering to remove additional background noise. Neural arches were manually removed from images. The cranial and caudal endplates were identified and manually attenuated.

To ensure that analyses were not biased due to differences in voxel size relative to bone size, image resolution was modified using integer coarsening to reduce variability in voxel aspect ratio and total number of voxels per specimen. For example, gibbon specimens were analyzed with a voxel size of 0.38 mm ×0.38 mm ×0.375 mm and human specimens were analyzed with a voxel size of 0.94 mm ×0.94 mm ×1.5 mm. A total of 11,535±4,348 voxels (mean ± SD) were used for each vertebral body and the aspect ratio (largest voxel dimension/smallest voxel dimension) ranged from 1.0–1.6. Finite element analyses of vertebral bones are not sensitive to voxel size across this range (assuming the aspect ratio remains small) [30]. The mineral density calibration phantom scanned with each specimen included five different solutions of known mineral density that are used to convert voxel brightness into mineral density (grams). Measures of bone mineral content (BMC, grams) and volumetric bone mineral density (vBMD, mg/cm^3^), vertebral body cross-sectional area (on the superior surface) and vertebral body volume were determined from the QCT scans as well.

**Figure 2:**
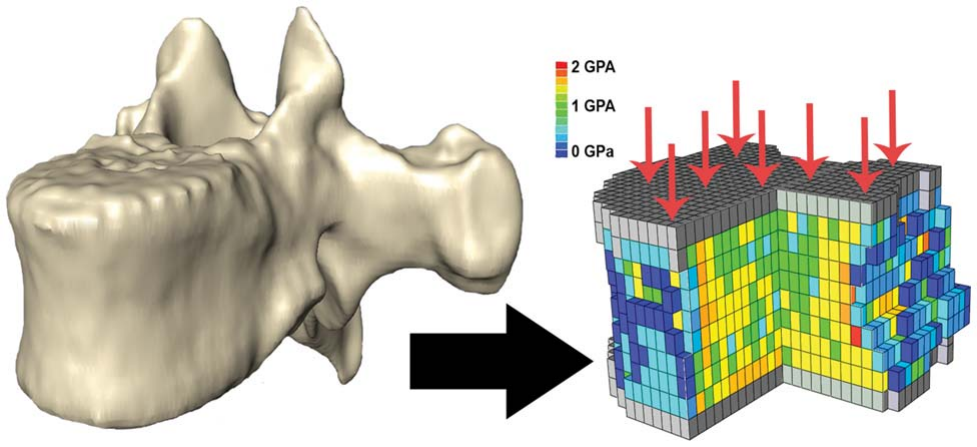
Biomechanical modeling to determine bone strength. (A) Three-dimensional images of vertebral bodies (a chimpanzee vertebra shown) were converted into finite element models for biomechanical analysis. (B) Finite element models were loaded in compression (arrows). Differences in the color of the bone elements represent different regional density and elastic modulus.

Finite element models of each vertebra were generated using a technique pioneered by Crawford and colleagues [29] (Please see [23] for a comparison of finite element modeling and previously used approaches). Briefly, each voxel in the image was represented in the finite element model as a transversely isotropic linear elastic 8-node brick element (Figure 2B). The elastic modulus of each voxel was determined by converting voxel brightness into mineral density using the mineral calibration phantom and subsequently converting mineral density to elastic modulus using an empirical relationship determined from human trabecular bone [23], [31]. The resulting elastic modulus values were binned into 50 different material properties to simplify finite element modeling. The superior surface of the model was assigned a uniform axial displacement corresponding to 3% deflection. A linear analysis using ABAQUS 6.8-3 (Dassault Systèmes Simulia Corp, Providence, Rhode Island, USA) was completed on 3.40 GHz Pentium 4 CPU with 2.0 GB of RAM. The compressive strength was derived from the whole bone stiffness determined in the finite element model using a simple column model as described by Crawford and colleagues [29]. Estimates of human vertebral bone compressive strength using this finite element modeling approach are highly correlated with compressive strength determined experimentally (r^2^>0.80) [29], [32].

### Trabecular Microarchitecture and Vertebral Shell Thickness

**Figure 3:**
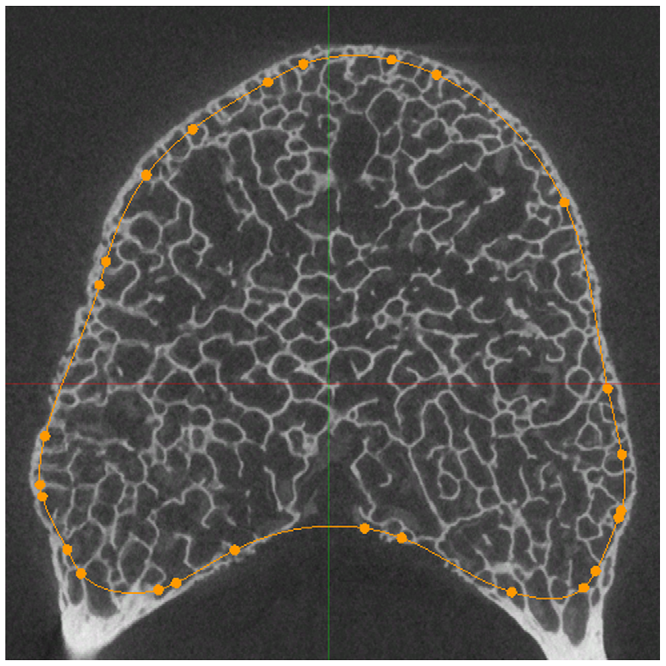
Delineation of vertebral shell and trabecular bone boundary. In transverse cross-sectional slices of the vertebrae, the boundary between the vertebral shell and the trabecular bone was traced. Here, an orange line denotes that boundary in a human vertebral body. Characteristics of the bone such as orientation of the bone and relative thickness of the shell and trabecular bone helped determine the placement of the boundary.

Microcomputed tomography images of vertebral bodies were collected for a subset of the specimens (n = 8–10 per species, Table 1). Images of the specimens were collected using a GE Locus eXplore RS micro-computed tomography machine (GE Healthcare, Milwaukee, WI, USA). Three-dimensional images of the each vertebra were collected with a voxel size of 46 µm. The vertebral shell and endplates were dissected digitally from each grayscale image by manual tracing in transverse slices (Spline ROI function in Microview Analysis+ 2.2, GE Healthcare, Milwaukee, WI, USA). The shell of the vertebral body is considered cortical bone; however, it is discontinuous (due to nutrient foramina) and can be similar in thickness to trabecular bone. Therefore, the vertebral shell was identified in transverse sections as the outermost, circumferentially oriented bone that demonstrated an increased density (Figure 3). To reduce labor associated with tracing, boundaries of the vertebral shell were made in slices one millimeter apart and the points between the slices were interpolated (Spline ROI function in Microview). Following interpolation, the images were checked for consistency and adjusted where appropriate. The whole vertebral body images were than separated to create two new images: one displaying only the shell only and the other displaying only trabecular bone.

**Figure 4:**
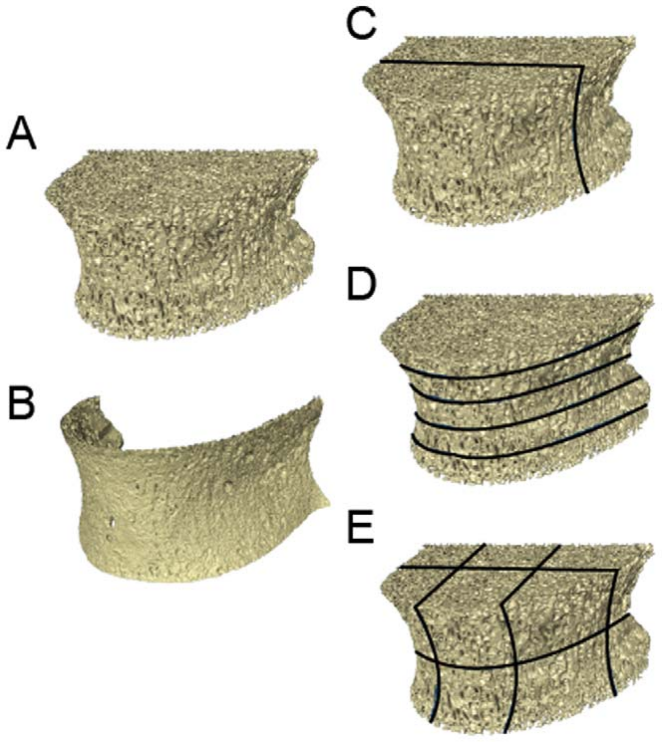
Analysis of trabecular microarchitecture. A micro-computed tomography image of a human vertebral body is shown. Images were divided into (A) trabecular bone and (B) vertebral shell. Variation in trabecular microarchitecture within the vertebral body was examined by considering variation in microarchitecture in (C) dorsal-ventral subregions, (D) transverse subregions and (E) across 12 anatomically determined subregions.

A custom program was written to threshold the images (separate bone from non-bone in a grayscale image) [24]. In addition to examining trabecular microarchitecture in the entire vertebral body, patterns in microarchitecture were explored by comparing microarchitecture in dorsal-ventral (two subregions), cranial-caudal (5 transverse subregions) and throughout the vertebra (12 subregions (Figure 4).

Traditional three-dimensional measures of trabecular microarchitecture (trabecular thickness, trabecular number, and degree of anisotropy) were achieved for the entire bone as well as each of the subregions using Quant3D (University of Texas-Austin) [33]. Trabecular thickness and number are determined using the distance transform method [34]. Degree of anisotropy describes the degree to which the trabecular structure has a preferential orientation and is determined using the mean intercept length method. Variability in trabecular bone density within vertebrae was measured as the inter-quartile range of bone volume fraction across entire vertebrae (using the 12 anatomically defined subregions). The inter-quartile range (IQR) across the 12 subregions was calculated as the difference between the third and first quartiles in each individual (the difference between the 4^th^ and 9^th^ most dense subregions). The inter-quartile ranges of the species were then compared using analysis of variance. The thickness of the vertebral shell was measured using measurement lines radiating from the dorsal center of the vertebral body [35]. One hundred lines were generated for each 0.046 mm thick slice. The mean of the thickness measurements was then determined for the entire shell and for each transverse subregion.

Statistical analyses were performed using MINITAB 15 (Minitab, Inc. State College, PA) with a significance threshold of α = 0.05. Analysis of variance was used to identify any differences in bone volume fraction and degree of anisotropy within species and among species. Regression analyses were performed to identify relationships between body mass and trabecular microarchitecture parameters, vertebral shell thickness, and/or vertebral body height. Regressions using body mass were performed using reduced major axis (RMA) to adjust for imprecision in body mass estimation [36]. For measures that were correlated with body mass, analysis of covariance implemented with a generalized least squares model was used to compare humans to apes including body mass as a covariate [37]. Post hoc Tukey multiple comparisons tests were used to determine any differences between species. No significant differences between the sexes were found after accounting for body mass, and consequently, all parameters were sex-pooled for analysis. The effects of phylogeny (evolutionary relationships among species) were not included in regression models as prior analyses of these species did not detect a significant effect of phylogeny and only extremely large phylogenetic influences can be observed in cohorts with fewer than seven species [23], [38].

## Results

**Figure 5:**
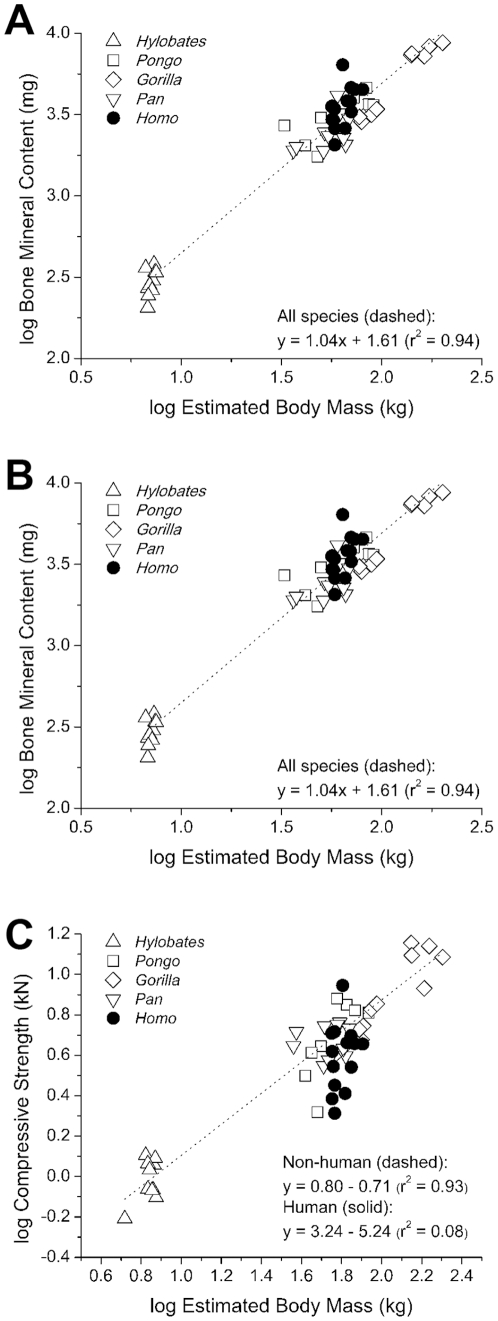
The relationships between body mass, bone mass and bone strength. (A) A positive correlation between body mass and bone mass (measured as bone mineral content) of the T8 vertebral body was observed that was similar in all species. (B) A positive correlation between bone mass and compressive strength of the T8 vertebral body was observed. Vertebrae from humans displayed reduced strength relative to bone mass (ANCOVA: p<0.01). (C) Although body mass was positively correlated with bone strength across species, vertebrae from humans showed reduced strength as compared to apes with similar body mass (ANCOVA: p = 0.04).

Average T8 density and size are shown in Table 2. After accounting for body mass human T8 vertebral bodies were significantly larger than those of apes in terms of vertebral height, cross-sectional area and volume (Table 3, p<0.05). Multiple comparisons among the species also support the idea that the human T8 is larger than each other species after accounting for body mass (no difference in cross-sectional area was observed between humans and orangutans, most likely due to the small sample size of orangutans and the conservative nature of the Tukey multiple comparisons test). Humans and apes have similar amounts of vertebral body bone mass relative to overall body mass (Figure 5A). However, vertebral body compressive strength relative to bone mass is less in humans than in the apes (p<0.01, Figure 5B). Additionally, humans display a reduced vertebral body compressive strength relative to body mass (p = 0.04 from ANCOVA, Figure 5C), indicating that human vertebrae are weaker (∼1.75 kN weaker) than those in apes with similar body mass. That humans have disproportionately low bone strength relative to bone mass, suggesting that fundamental differences in bone structure exist between humans and apes.

**Table 2:** Measures of young adult vertebral body dimensions and density in humans and apes are shown (n = 8–14 per species, Mean ± SD).

	Gibbon	Orangutan	Gorilla	Chimpanzee	Human
Body Mass (kg)	6.91±0.65	58.80±15.68	123.99±45.44	55.26±11.29	64.20±7.52
Bone Mineral Density (from QCT scan, g/cm^3^)	360.10±43.25	340.53±49.21	368.20±42.33	386.24±27.07	279.33±43.57*
Bone Mineral Content (from QCT scan, g)	0.30±0.06	3.16±0.95	5.28±2.40	2.49±0.71	3.59±1.05
Vertebral Body Height (mm)+	8.51±0.35	16.35±1.17	17.01±2.22	14.34±1.07	19.8±1.27
Vertebral Body Cross-sectional area (cranial endplate, mm^2^)+	79.30±12.10	460.52±116.18	692.80±222.80	355.50±49.70	566.72±95.24
Vertebral Body Volume (mm^3^)+	677.00±121.00	7599.62±2232.45	12147.00±5093.00	5137.00±1054.00	12735.88±2394.19

+ See Table 3 for analysis of covariance accounting for differences in body mass.

*Significantly different from all other species (p<0.05).

**Table 3:** Analysis of covariance is used to compare measures of vertebral bodies with body mass as a covariate (n = 8–14 per species).

		Vertebral Body Height (mm)				Vertebral Body Cross-sectional Area (mm^2^)			Vertebral Body Volume (mm^3^)		
Human v. Non-Human		p<0.01				p<0.01			p<0.01		
**Multiple Comparisons** *	**Hylobates**	A				A			A	B	
	**Pongo**		B				B	C	A		
	**Gorilla**			C		A	B			B	
	**Pan**		B	C		A	B			B	
	**Homo**				D			C			C

*Multiple comparisons among all species are shown such that species that do not share a letter are significantly different from one another (p<0.05, Tukey post-hoc).

**Figure 6:**
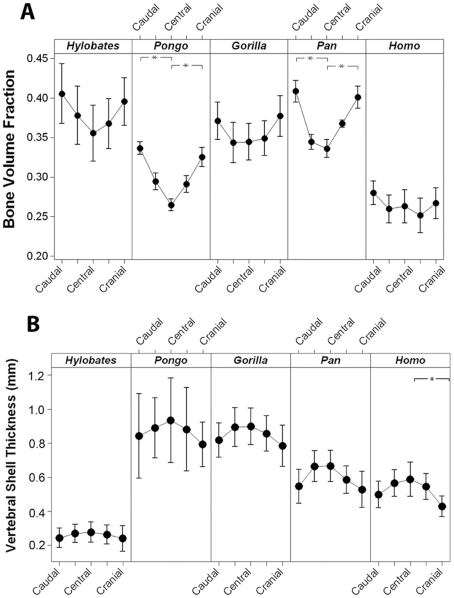
Cranial-Caudal variation in trabecular bone volume fraction and shell thickness. Interval plots displaying mean (filled circle) and 95% confidence interval (bars) of (A) bone volume fraction and (B) vertebral shell thickness corresponding to the five transverse subregions of the vertebral bodies are shown. A line connects the means symbols to better visualize the trend in bone volume fraction within the vertebral bodies of each species. Stars indicate subregions that are significantly different within species (p<0.05). Humans displayed a reduced overall bone volume fraction as compared to apes (p<0.01).

With regard to internal structure, trabecular bone volume fraction (BV/TV) was significantly lower in humans (Table 4, p<0.05). Thus, human vertebrae are more porous than ape vertebrae in this young age group. Among the apes, gibbons, gorillas and chimpanzees have a significantly higher bone volume fraction than orangutans (Figure 6A, p<0.05). No other significant differences in trabecular microarchitecture of the whole vertebra were observed among species (Table 4). Trabecular bone volume fraction was not significantly related to body mass. Trabecular thickness (Tb.Th) was correlated with bone strength among species (p<0.05). No other measures of trabecular microarchitecture were correlated with bone strength.

**Table 4:** Measures of trabecular microarchitecture in the young adult vertebral body are shown for humans and apes (n = 8–10 per species, Mean ± SD).

	Gibbon	Orangutan	Gorilla	Chimpanzee	Human
Bone Volume Fraction (BV/TV)	0.43±0.12	0.30±0.01	0.36±0.06	0.37±0.02	0.25±0.04*
Degree of Anisotropy	1.76±0.11	1.54±0.10	1.58±0.19	1.73±0.14	1.78±0.20
Trabecular Thickness (Tb.Th)	0.16±0.02	0.21±0.03	0.22±0.05	0.18±0.03	0.17±0.02
Trabecular Number (Tb.N)	2.87±1.09	1.47±0.19	1.66±0.30	2.13±0.35	1.52±0.13
Inter-Quartile Range in Bone Volume Fraction (×10^−2^)^+^	4.80±0.70	5.21±3.60	5.20±3.50	4.43±1.20	3.06±0.80
Differences in Degree of Anisotropy (Dorsal-Ventral)	0.33±0.18#	0.04±0.21	0.14±0.32#	0.37±0.23#	0.45±0.31#
Mean Vertebral Shell Thickness	0.26±0.05	0.87±0.17	0.86±0.15	0.60±0.11	0.52±0.10

+ Inter-quartile range is determined using measures of bone volume fraction in the 12 anatomically defined subregions shown in Figure 3E.

*Significantly different from all other species (p<0.05).

# Significantly greater than zero (p<0.05).

Only subtle differences in distribution of trabecular bone mass were present among species. No difference in bone volume fraction was found between ventral and dorsal subregions among species; however, within species, differences in the orientation of the trabecular microarchitecture were observed between the ventral and dorsal subregions. With the exception of orangutans, hominoids possessed a significantly higher degree of anisotropy in the ventral subregions than in the dorsal subregions (orangutans, p = 0.60, all others, p<0.05). Among the transverse subregions, all species displayed a trend with greater trabecular bone volume fraction closer to the endplates and lower trabecular bone volume fraction near the center of the vertebral body (Figure 6A). Shell thickness appeared to increase toward the midtransverse plane of the vertebral body (Figure 6B), presumably compensating for the decrease in trabecular bone volume fraction. No differences in inter-quartile range in bone volume fraction were observed among species (Table 4). Among species, no other significant differences or obvious trends in variability in microarchitecture within vertebrae were observed. No significant relationships between bone strength and within-vertebra variability in microarchitecture were observed.

**Figure 7:**
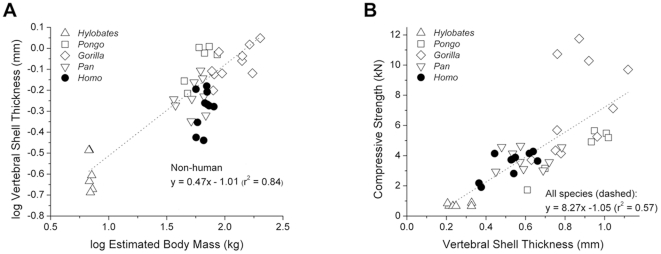
The relationships between shell thickness, body mass and whole bone strength. (A) Vertebral shell thickness was positively correlated with body mass among the species but humans have thinner vertebral shells relative to body mass (ANCOVA: p<0.01). (B) A positive correlation between compressive strength and vertebral shell thickness was shown. No differences were observed among species.

Mean thickness of the vertebral shell was positively correlated with body mass; however, humans have thinner vertebral shells than would be expected for their body mass (p<0.01) (Figure 7A). Vertebral shell thickness was positively correlated with compressive strength across all species (p<0.01), and the relationship between shell thickness and compressive strength was similar among all species (Figure 7B).

## Discussion

While age-related bone loss is a major factor determining risk of spinal fracture in older humans, the development of osteoporosis is more sensitive to peak bone mass and strength achieved at adulthood [14]. We found that young adult human vertebrae have reduced strength as compared to young adult apes with similar bone mass and body mass. The relatively low bone strength in human vertebral bodies is caused by reduced vertebral trabecular bone volume fraction and a thinner vertebral shell. Our findings demonstrate biomechanical and structural differences in vertebrae between humans and apes are present prior to the onset of age-related bone loss; given the association between peak bone mass and risk of osteoporosis mentioned above, the observed differences among species likely contribute to the unique susceptibility of humans to vertebral fractures later in life.

The distribution and orientation of trabecular bone within the vertebral bodies was more similar among the extant hominoids than was expected given the differences in primary locomotor repertoires of the apes (brachiation, careful-climbing, knuckle-walking). The increased degree of anisotropy and cranio-caudal orientation of the trabecular bone in the ventral subregions of the vertebral bodies reflects ventral and dorsoventral flexion and extension of the thoracic spine seen in primates [39], [40]. Additionally, the trabecular alignment is consistent with electromyography studies of ape back muscles that indicate that the kinematics and loading of the spinal column is similar among these species [41].

Humans have a thinner vertebral shell than apes after accounting for body mass. The thickness of the vertebral shell has been shown to play an important role in determining vertebral bone strength within humans [42]. When age-related bone loss occurs, the load sharing relationship between the shell and cancellous bone is altered such that more load is carried by the vertebral shell, possibly influencing fracture risk within humans [43]. The thicker shells in ape vertebrae may make the bones more resistant to fracture even after age-related bone loss.

The current study is the first that we are aware of to combine morphological descriptions of human and ape vertebrae with both finite element modeling and analysis of internal microarchitecture. The biomechanical and microstructural modeling techniques are commonly applied to human bones in the medical literature and provide new ways of contrasting human and ape vertebrae. Additionally, the study included a relatively large number of rare, complete ape and human skeletons, allowing confirmation of adulthood and estimation of body mass from non-vertebral bones.

There are some limitations that must be considered while interpreting our findings. First, the goal of the study was to understand differences in susceptibility to fracture among species and not fracture risk within individuals. It is important to keep in mind that traits differing *among* species are not necessarily traits that would indicate fracture risk *within* a species. That being said, humans have reduced bone volume fraction and reduced vertebral shell thickness as compared to apes and those traits are believed to influence fracture risk *within* humans. Second, the biomechanical analysis assumed that the relationship between trabecular bone density and Young's modulus was the same among species, an assumption that is reasonable given the close phylogenetic relationship among the species examined [18] and the similar microstructure reported in the current study. Third, the current study addressed humans and apes due to their close phylogenetic relationships and it is unclear to what degree our findings may be applied to other primates. While small numbers of vertebral fractures of unknown etiology have been observed in a free-ranging colony of rhesus macaques, the fractures were not associated with osteopenia and further research is needed to determine if these fractures were caused by traumatic falls from trees in this partially arboreal species [44].

Prior work has suggested that contemporary humans are not as physically active as wild apes (or early hominids) and that humans have reduced bone density and strength as a result of inactivity [45]. Habitual loading on vertebrae is difficult to compare among species; however, reduced physical activity is not required to explain the reduced bone volume fraction and strength we observed in human vertebrae. The increased cross-sectional area of human vertebral bodies allows load to be distributed across a larger area, leading to a reduction in habitual tissue stress of the underlying bone, a condition that would be expected to cause bone loss due to reduced tissue stress. Hence, even if the amount of physical activity and magnitude of habitual loading in humans were the same as that in wild apes (relative to body mass), humans would still be expected to have reduced vertebral bone volume fraction (i.e. more porous vertebrae) because the habitual loading is distributed across a larger vertebral bone surface area. Given the more pronounced thoracic kyphosis in humans, we speculate that habitual loading on the 8^th^ thoracic vertebra is actually greater (relative to body mass) than in apes, suggesting that reduced vertebral bone volume fraction may be caused by factors other than functional adaptation. Additionally, while contemporary humans may be less active than past populations, osteopenia has been found in medieval skeletal populations that were presumably more active than individuals in industrialized societies [46]–[48].

Lastly, nutritional history plays an important role in the development of osteoporosis within humans and food intake differs considerably among the species examined. While it is unlikely that apes in the wild receive better nutrition than the modern human population, aspects of agricultural-based diet of contemporary humans have been implicated as a factor contributing to the reduced bone mass in modern humans [49]. While nutrition may contribute to differences among species, it is unlikely to explain the increased volume, height and cross-sectional area of the human vertebral bodies as compared to apes as these traits are part of a suite of musculoskeletal adaptations to bipedalism [9], [50]–[52]. Recent studies suggest that early hominins (*Australopithecus* and Neanderthals) also display disproportionately large vertebral bodies as compared to extant apes [53], suggesting that other bipedal primates that did not enjoy contemporary human diets also display the observed trends in external vertebral structure. Clearly the increased vertebral cross-sectional area in humans cannot be explained by differences in nutrition alone nor can nutrition explain the observed differences in vertebral bone strength and morphology between modern humans and apes.

We propose instead that the increase in volume and reduction in bone volume fraction and shell thickness of the human vertebral body is a byproduct of the evolutionary development of habitual bipedalism. The adoption of bipedality in the human lineage required a systemic reorganization of the musculoskeletal system from a quadrupedal ancestor. While there are substantial benefits to bipedality that improve Darwinian fitness, this reorganization resulted in novel upright postures and striding gait that could compromise the functioning of the locomotor skeleton. In human bipedalism, the calcaneus and lower limb joints experience relatively high impact loads during the heel strike phase of the gait cycle as compared to quadrupedal primates [54]. The joint surfaces of the human lower limb therefore display an increase in size that allows for forces during heel strike to be distributed across a larger area, maintaining a healthy distribution of mechanical stress within the cartilage and bone [18], [50]. Additionally it has been proposed that increased porosity in the calcaneus, distal femur and proximal tibia in humans can increase energy absorption by these bones [9], [18]. The increase of the cranial and caudal surfaces of the vertebral body is likely analogous to the disproportionate increase in size of the synovial joints of the human hindlimb due to bipedalism, and may very well be secondary to the evolutionary adaptations in the lower limbs. It has been shown that joint development in the limbs and the spine is controlled by many of the same developmental signaling factors [51], [55], [56]. We speculate that increased vertebral body size and porosity may very well be a systemic adaptation to bipedalism as evidenced by increased joint size of the distal femur and proximal tibia and the increased size and porosity of the human calcaneus (as compared to apes) [9], [18], [50], [52], [57], [58].

As with many other age-related maladies in humans, the reduced strength of the vertebrae was not subjected to negative selection pressure in ancient hominins because it had relatively little effect on reproduction, since spinal fractures do not develop until well past the child-rearing years [10]. Contemporary humans have a longer lifespan, however, such that age-related bone loss exacerbates the already more porous human vertebrae enough to make humans susceptible to vertebral fracture. While apes exhibit age-related bone loss, they do not experience spontaneous vertebral fractures because their bone strength in early adulthood is much greater, theoretically requiring more bone loss before spinal fragility fractures would be observed. While there are many different contributors to the development of osteoporosis within individuals, the capacity of humans to experience vertebral fracture appears to be a byproduct of our unique systemic adaptations to bipedality.
